# A Survey on Sleep Disorders and Related Hormones in Patients with Newly Diagnosed Systemic Lupus Erythematosus

**DOI:** 10.31138/mjr.32.2.148

**Published:** 2021-06-30

**Authors:** Maryam Sahebari, Sahar Ravanshad, Yalda Ravanshad, Fariborz Rezaeitalab, Houshang Rafat Panah Bayegi, Hadi Asadpour, Seyed Alireza Javadinia, Zahra Rezaieyazdi

**Affiliations:** 1 Rheumatic Diseases Research Center, Mashhad University of Medical Sciences, Mashhad, Iran; 2 Department of Internal Medicine, Faculty of Medicine, Mashhad University of Medical Sciences, Mashhad, Iran; 3 Education Development Center, School of Medicine, Mashhad University of Medical Sciences, Mashhad, Iran; 4 Department of Community Medicine, Mashhad Branch, Islamic Azad University, Mashhad, Iran; 5 Department of Neurology, School of Medicine, Mashhad University of Medical Sciences, Mashhad, Iran; 6 Immunology Research Center, Division of Inflammatory Disease, Mashhad University of Medical Sciences, Mashhad, Iran; 7 Sleep Clinic of Ebn-e-Sina Hospital, Psychiatry and Behavioral Sciences Research Center, School of Medicine, Mashhad University of Medical Sciences, Mashhad, Iran; 8 Student Research Committee, School of Medicine, Mashhad University of Medical Sciences, Mashhad, Iran

**Keywords:** Systemic lupus erythematosus, sleep disorders, polysomnography, sleep, melatonin, prolactin

## Abstract

**Background and Objectives:**

Systemic lupus erythematosus (SLE) is reportedly associated with sleep disorders. Thus, the present study aimed to investigate sleep disorders in newly diagnosed SLE patients.

**Materials and Methods:**

This study was conducted on patients with newly diagnosed SLE (ie, case group) and a control group. The case and control groups were matched in terms of gender, age, socioeconomic status, and educational level. Venous blood samples were obtained from the participants to measure prolactin and melatonin levels. Furthermore, they were subjected to polysomnography. The data were analysed by SPSS (version 16) at a significance level of 0.05.

**Results:**

A total of 28 women were enrolled in this study (ie, 14 individuals in each group). The frequencies of sleep disorder in the case and control groups were obtained as 64.3% and 50%, respectively (P=0.4). These two groups had the mean sleep onset times of 10.76±10.64 and 8.67±7.12 min (P=0.5) and the respiratory disturbance indices of 9.20±10.23 and 8.44±9.27, respectively (P=0.8). The frequency of sleep apnoea was obtained at 50% for both case and control groups (P=1). There was no significant difference between these groups in terms of the mean serum prolactin and melatonin levels (P=0.3 and P=0.2, respectively). Serum melatonin level showed a direct correlation with sleep latency to N1 (i.e., the first part of non-rapid eye movement in sleep) and spontaneous arousal index in the case group (P=0.02, r=0.602 and P=0.04, r=0.544, respectively).

**Conclusion:**

According to the findings, there was no significant difference in the frequency of sleep disorders between the healthy subjects and patients at the onset of lupus. Additionally, melatonin and prolactin levels showed no significant difference between the groups. Our results are inconsistent with previous studies, due to the difference in disease duration probably. It seems that the chronicity and complications of the disease, as well as the adoption of glucocorticoid therapy for the chronic disease affect sleep quality in SLE patients more than disease duration.

## INTRODUCTION

Systemic lupus erythematosus (SLE) is an autoimmune disease with an unknown aetiology that affects different organs and presents with different clinical symptoms. The chronic and debilitating nature of SLE, as well as the pathogenesis of the disease itself, is associated with the likelihood of developing psychiatric and neurological problems, such as mood, anxiety, and sleep disorders.^1,2^ Sleep is an active and complex state, associated with significant changes in physiology, including muscle tone, body temperature, endocrine system, and gastrointestinal and cardiovascular activities.

Sleep is subdivided into two stages, including rapid eye movement (REM) and non-REM (NREM).^3^ The REM phase happens progressively in wake-up time. Sleep-related hormones are cortisol, prolactin, melatonin, and thyrotropin.^4^ Melatonin is synthesised in the pineal gland during the night and secreted to blood and cerebrospinal fluid. This substance induces sleep in brain of humans and other mammals and has effect on the various phases of sleep. Melatonin secretion level decreases during daylight hours, peaks after darkness (ie, between 11 pm and 3 am), and then drops rapidly before daylight, in all mammals.^5^

Prolactin is secreted from the pituitary gland, which regulates immunity and triggers autoimmunity. Hyperprolactinemia occurs periodically during the sleep. However, prolactin returns to normal level during the first hours of awakening. Sex hormones, especially oestrogen and prolactin, play an important role in regulating the immune response.^6^

The prevalence of sleep disorders and the relevant factors to the quality of sleep have yet to be defined clearly in SLE patients, although studies have shown these kind of impairments are remarkably common (over 80%) among them.^7,8^ Sleep problems challenge the management of SLE due to creating disturbance to the patient’s quality of life, increasing fatigue, depression, absenteeism, and healthcare costs.^7–9^ Polysomnography, along with several sleep rating systems, such as the Pittsburgh Sleep Quality Index (PSQI), are used to investigate sleep disorders.^10^ Recognition of sleep patterns and identification of sleep disorders in patients with SLE, as well as research on the affective hormones on sleep, can help to decrease sleep-related issues and improve their quality of life by implementing proper interventions. In this regard, the present study was conducted to investigate sleep disorders in patients with newly diagnosed SLE. The main research question in this study was that, if reported sleep disorders in patients with newly diagnosed SLE are related to the disease progression, or secondary to disease and complications.

## MATERIALS AND METHODS

This cross-sectional case-control study was conducted on adult patients with newly diagnosed SLE referring to the Rheumatic Diseases Research Centre in accordance with the recommendation of the American College of Rheumatology (2012) during 2016.^2^ The exclusion criteria were: 1) history of other systemic diseases, 2) history of the corticosteroid therapy, 3) consumption of psycho-tropic or hypnotic drugs before entering the study, 4) pregnancy or lactation, and 5) use or abuse of opioids. The control group was selected from the eligible healthy volunteers by doing physical examination and an interview with patient relatives (preferably first-degree ones), who are in similar socioeconomic status. In terms of gender and age, these individuals were homogenous with the case group as matched pairs. All patients and controls were interviewed about recent emotional distress or important changes in their life; participants with positive history were removed from the study. All the participants were informed about the methodology and objectives of the study. Written informed consent was taken prior to any interference and implementation. The study was approved under the ethics code of IR.MUMS. fm.REC.1394.143. The patients with a SLE disease activity index (SLEDAI2k) of > 4, completed a checklist covering demographic information and sleep history, to self-report on their sleep history (**Table 4**).

In order to measure prolactin and melatonin levels, 5cc brachial vein blood samples were taken from all patients at 8:00PM before the sleep test. The blood samples were immediately centrifuged to separate the serum, and then stored at –20°C until testing. The evaluation of melatonin and prolactin levels were accomplished by applying the Eliza Kit for Melatonin (ZellBio GmbH, IBL international GmbH, Ulm, Germany) and Elecys® Prolactin Kit (Roche Diagnostics IT Solutions GmbH, Switzerland), respectively.

As to prevent the onset of physiological hyperprolactinaemia, all the participants were advised to avoid nipple stimulation and orgasm within a week before sampling. As for laboratory parameters, C3 ratio was calculated by dividing patient’s C3 by the lower limit of normal (LLN) C4 ratio was obtained by dividing patient’s C4 by the LLN, and anti-DNA ratio was estimated by dividing patient’s anti-DNA to the laboratory reference range.

The patients underwent polysomnography, including simultaneous monitoring of electroencephalogram, electrooculogram and electrocardiogram, in the sleep lab of Ebn-e-Sina Hospital.^1^ Respiratory movements were measured by utilising plethysmography bands. In addition, a four-bead thermistor or a system equipped with a sensor was used to record the airflow through the nose or mouth. Oxygen saturation was also determined using pulse oximetry. The patient, monitoring device, and the corresponding technician were in separate rooms. The patient’s room was comfortable, well-ventilated, and sound-proofed. A day before polysomnography test, an appointment was arranged as to accustom the patient to the environment. The patients were informed about needed preparations for evaluation in the meeting. Clearly, they were required to have a calm day without nap, take a bath, and have a light meal. The data were obtained for one night. An experienced technician performed the quantitative assessment of the sleep stages through observation according to the standard guidelines, which has been defined by the American Academy of Sleep Medicine.^11^ In brief, normal sleep is divided into NREM and REM sleep. The NREM sleep is further divided into progressively deeper stages of sleep; namely, stage N1, N2, and N3 (ie, deep or delta-wave sleep). In sleep science, sleep onset latency is the length of time that it takes to accomplish the transition from full wakefulness to sleep, normally to the lightest of the non-REM sleep stages.

### Statistical analysis

The data were analysed by applying SPSS software (version 16). The quantitative and qualitative variables were presented as percentage, frequency, mean, and standard deviation, by using frequency distribution tables. Fisher’s exact test was applied to compare the demographic data to the quality of sleep variables. Furthermore, the comparison of different indicators of sleep disorder was performed by using t-test and Mann-Whitney U test. Moreover, the Pearson correlation coefficient was used to investigate the relationship between sleep and serum levels of prolactin and melatonin. Finally, P-value less than 0.05 was considered statistically significant.

## RESULTS

A total of 28 females were enrolled in the study (ie, 14 cases in each group). The case and control groups were matched in terms of age (37.93±11.80 and 39.43±11.81 years, respectively=0.7), marital status (marriage frequency of 64.3% and 71.4%, respectively P=1), and educational level (≥high school frequency of 100% and 80.7%, respectively; P=0.5). The mean body mass index (BMI) in the study population (16–40 kg/m^2^) was 24.06±5.60 kg/m^2^. Moreover, the case and control groups had the mean BMIs of24.47±6.62 and 23.65±4.57, respectively (t=0.034, P=0.9).

Based on the systemic lupus erythematosus disease activity index (SLEDAI)-2K scoring system, the severity of lupus disease in our patients was estimated as 15.71±3.49.

The mean of the interval between clinical presentation and diagnosis was 38±10 days. Given that all patients were newly diagnosed, they all had a SLEDAI score of > 4; accordingly, the disease was in the active phase in all cases. The most common clinical symptoms in the patients with lupus were arthritis (71.4%), rash (64.3%) and oral ulcer/alopecia (50%). In this regard, fever had a frequency of 21.4%. Among the laboratory evaluations, the most commonly used test was disrupted leukopenia (42.9%). There was no case with reduced level of platelets or proteinuria. The most prevalent immunologic impairment in the patients with lupus was positive antinuclear antibody (98.8%) followed by elevated anti-double stranded DNA (anti-dsDNA) (78.6%), positive anti-SSA (42.9%), and anti-ribonucleoprotein antibody (35.7%).

Sleep disturbance in the case and control groups had a frequency of 64.3% (n=9) and 50% (n=7) respectively. Based on the results of Fisher’s exact test, there was no statistically significant difference between these two groups (P=0.4). Regarding the sleep-related variables, there were no significant difference between these groups as presented in **Table 1** and **Table 2**. The frequency of sleep apnoea was 50% (n=7) in both groups (P=1) (**Table 2**), and there is no significant correlation between sleep items and SLEDAI (**Table 3**).

**Table 1.** Sleep quality variables in participants.

**Sleep items**	**Classification**	**Case group**		**Control group**		**Statistical test**
		**Frequency**	**Percentage**	**Frequency**	**Percentage**	
Problem in sleeping	Yes	2	14.4	0	0	P=0.1^†^ Phi=0.400
	Somewhat	6	42.8	3	21.5	
	No	6	42.8	11	78.5	
Excessive daytime sleepiness	Yes	4	28.5	1	7.2	P=0.3^†^ Phi=0.324
	Somewhat	4	28.5	3	21.5	
	No	6	42.8	10	70.5	
Fatigue	Yes	6	42.8	4	28.5	P=0.1^†^ Phi=0.376
	Somewhat	3	21.5	8	57.1	
	No	5	35.7	2	14.4	
Impaired concentration	Yes	3	21.5	4	28.5	P=0.5^†^ Phi=0.230
	Somewhat	6	42.8	3	21.5	
	No	5	35.7	7	50	
Awakening at night	More than three times	4	28.5	1	7.2	P=0.1^†^ Phi=0.417
	One to two times	8	57.1	6	42.8	
	Not waking up	2	14.4	7	50	
Time of waking up in the morning		1	7.2	1	7.2	P=0.7^†^ Phi=0.152
		4	28.5	6	42.8	
		9	64.3	7	50	

† Fissure’s exact test.

**Table 2.** Comparison of polysomnographic items between lupus patients and healthy controls.

**Polysomnographic items**	**Case group mean±SD**	**Control group mean±SD**	**P-value**
Sleep latency(minutes)	10.76±10.64	8.68±7.12	P=0.5†† t=0.607
Night-time sleep duration (hours)	7.5±1.4	7.5±1.7	P=1†† t=0
Frequency of awakenings during sleep	15.07±7.34	19.78±8.30	P=0.1†† t=–1.59
Wake-up hour in the morning (hours)	7.28±1.9	6.89±1.6	P=0.6†† t=0.582
Respiratory disturbance index	9.20±10.23	8.44±9.27	P=0.8†† t=0.205
Total arousal index	13.93±11.93	12.45±7.80	P=0.7†† t=0.388
Oxygen desaturation events	3.33±4.36	5.1±6.88	P=0.4†† t=–0.802
Stage R latency from sleep onset (minutes)	106.54±46.33	127.75±67.60	P=0.3†† t=–0.969
Wake after sleep onset	58.42±66.53	61.31±37.21	P=0.8†† t=–0.142
Sleep efficacy	16	13	P=0.4# z=0.965
Central sleep apnoea	13.71	15.29	P=0.6# z=0.555
Spontaneous arousal index	15	14	P=0.8# z=1
Periodic leg movement arousal index	14.5	14.5	P=1# z=0.0
Periodic leg movements sequence index	12.93	16.07	P=0.3# z=1.2

† Fissure’s exact test;

†† t-test;

# Mann-Whitney U test.

**Table 3.** Relationship between sleep items and SLEDAI.

**Polysomnographic items**	**SLEDAI p(r)**
Sleep latency	0.7 (–0.130)
Total arousal index	0.5 (–0.174)
Respiratory disturbance index	0.9 (–0.459)*
Central apnoea	0.6 (0.125)
Stage R latency from sleep onset	0.7 (0.115)
Desaturation index	0.3 (–0.276)
Number of awakenings	0.6 (0.112)
Sleep efficiency	0.7 (–0.151)
Spontaneous arousal index	0.5 (0.188)
Periodic leg movements sequence index	0.8 (–0.069)

* Analysis by Pearson test was significant at the significance level of P≤0.05. All analyses in this table were performed by Pearson test.

The results confirmed that, the case and control groups had the mean serum prolactin levels of 10.70±18.02 and 14.57±8.40 ng/ml (t=0.9, P=0.3), and mean serum melatonin levels of 142.65±226.72 and 316.57±350.07 pg/ml, respectively (t=–1.32, P=0.2). Investigation of the correlation between prolactin level and quantitative sleep items revealed that this serum level was not associated with any of the sleep items in patients. However, melatonin level showed a significant positive correlation with sleep latency to N1 and the spontaneous arousal index; SAI: the number of arousals and awakenings is registered in the study and reported as a total number and as a frequency per hour of sleep, which is referred to as an index. The higher the arousal index, the more tired you are likely to feel, (although people vary in their tolerance of sleep disruptions), in the case group (r=0.602, P=0.02 and r=0.544, P=0.04, respectively).

However, there was no significant relationship between the melatonin level and any of the sleep items in the control group. Furthermore, serum prolactin and melatonin levels demonstrated no significant correlation with SLEDAI scores (r=0.020P=0.43 and r=0.203, P=0.4, respectively). Prolactin level had no significant correlation with anti-dsDNA ratio (P=0.6), C3 ratio (P=0.1), and C4 ratio (P=0.6). The serum prolactin level was positively associated with the serum melatonin level in all patients; however, this relationship was not statistically significant (r=0.1, P=0.5). Additionally, no significant correlation was observed between prolactin and melatonin levels in the case and control groups (r=0.41, P=0.1 and r=0.06, P=0.8, respectively) (**Table 4**).

**Table 4.** Relationship between sleep items and serum levels of prolactin and melatonin in each research group.

	**Serum prolactin levels p(r)**		**Serum melatonin level p(r)**	
**Polysomnographic items**	**Patient**	**Control**	**Patient**	**Control**
Sleep latency	0.6 (–0.162)	0.3 (–0.327)	0.3 (+0.320)	0.6 (+0.142)
Total arousal index	0.6 (–0.142)	0.9 (–0.144)	0.2 (–0.382)	0.8 (–0.069)
Respiratory disturbance index	0.6 (–0.138)	0.4 (–0.240)	0.7 (–0.133)	0.1 (–0.457)
Central apnoea	0.3 (+0.313)	0.3 (–0.292)	0.9 (–0.002)	0.6 (–0.167)
Desaturation index	0.4 (–0.230)	0.2 (–0.357)	0.4 (–0.267)	0.6 (–0.162)
Number of awakenings	0.2 (–0.330)	0.5 (–0.184)	0.6 (–0.145)	0.3 (–0.284)
Sleep efficiency	0.2 (+0.376)	0.2 (+0.398)	0.6 (+0.146)	0.5 (+0.217)
Spontaneous arousal index	0.9 (0023)	0.3 (0.035)	0.04 (+0.544)^*^	0.4 (0.43)
Periodic leg movements sequence index	0.06 (0.514)	0.2 (–0.347)	0.8 (+0.078)	0.6 (–0.169)
Sleep latency to N1	0.7 (–0.099)	0.7 (–0.092)	0.02 (+0.602)^*^	0.07 (+0.501)
Sleep latency to N2	0.5 (–0.183)	0.4 (–0.266)	0.07 (+0.490)	0.9 (+0.021)
Sleep latency to N3	0.5 (–0.171)	0.2 (–0.352)	0.1 (+0.388)	0.8 (–0.091)
Stage R latency from sleep onset	0.3 (–0.316)	0.5 (–0.186)	0.5 (–0.173)	0.3 (+0.329)

* Analysis by Pearson test was significant at the significance level of P≤0.05. All analyses in this table were performed by Pearson test.

## DISCUSSION

This study was conducted to investigate the status of sleep disorders in patients with newly diagnosed SLE compared to the control group. The results indicated there is no significant difference between the SLE patients and control groups regarding the polysomnographic findings. In addition, the serum levels of prolactin and melatonin were not significantly different between the mentioned groups. Investigating of the sleep items and SLEDAI revealed that there is no relationship between disease activity (SLEDAI) and the polysomnographic indices. In terms of the quantitative sleep-related items, sleep latency to N1 and the SAI showed a significant positive correlation with serum melatonin level in the case group. Overall, the present results indicated that sleep disorders were significantly prevalent in both patients with lupus and control subjects. There was no significant difference between these groups. Besides, there was not any correlation between sleep disorders and disease activity in lupus. According to **Table 5**, the application of polysomnography in the diagnosis of sleep quality disorders in these patients is the main superiority of the present study over other similar studies that have used the questionnaire. The prevalence of sleep disorder was similar to the previous studies (over 60%). A review study performed in 2014^7^ revealed that over 80% of patients suffered from sleep disorders and low quality of sleep, which were associated with disease activity. In the mentioned study, the primary sleep disorders among the patients with lupus included higher sleep latency, sleep fragmentation, increased number of arousals, increased awakening number during the night, and decreased sleep efficiency. In contrast, a number of studies have not observed such relationships.^12^ These discrepancies between them can be judged due to the differences in the population size of the studies, stage of the disease, and ethnicity. The most important reason of this disagreement is ascribed to the characteristics of the patients. Kotb et al. (2013) demonstrated that sleep disorders were significantly associated with the duration of illness, disability, severity of pain, organ damage, and depressed mood.^13^ Tufik and Palma (2009) conducted a similar study on animal models of mice with lupus. They reported that sleep disturbances in the affected group increased as the disease progressed.^14^

**Table 5.** Comparison of the results of the present research with those of other studies regarding sleep disorders in lupus disease.

**Author name**	**Research units (n)**	**Methodology of assessing sleep disorders**	**Relationship of sleep disorders and SLEDAI**
Valencia-Flores (2010)^9^	• Diagnosed patients, outpatients, all patients under treatment (n=40) • Healthy women (n=25)	Night polysomnogram	• SLE patients had less sleep efficiency (85.9%±7.0 versus 90.9%±3.5; P=0.002), more frequent waking-up (8.1±4.1 and 4.9±2.1; P=0.002), higher mean PML (5.3±8.4 and 1.6±1.7%; P=0.04), of the lower mean total sleep time (391.0±35.3 and 426.2±25.1 min; P=0.001)
Kasitanon (2013)^10^	Diagnosed patients, outpatients (n=56), 28 people under treatment • No control	PSQI	• SLEDAI was 4.32±5.22 in subjects with normal sleep and 4.16±4.47 in subjects with sleep disturbances, which was not significant even after regression analysis. • Factors affecting the sleep disorders were psychological disorders, depression, anxiety, and pain.
Vina (2013)^12^	• Diagnosed patients, outpatients, all patients under treatment (n=118) • General population as control group	Medical Outcomes Study Sleep Scale	• Sleep disturbance relationship with SLEDAI was not significant after regression analysis. • An effective factor in sleep disorder was depression.
Rady (2013)^13^	• Diagnosed patients, outpatients, all patients under treatment (n=30) • Healthy women (n=30)	PSQI	• Global PSQI was 8.47±3.53 in the case group and 5.10±3.66 in the control group, which was significantly different in all PSQI subunits (P=0.0001). • The sleep quality was associated with disease activity (t=0.695, P=0.000) •SLEDAIs of the patients with poor sleep and good sleep were 13.96±4.79 and 6.29±2.14, respectively (P=0.001).
Gholamrezaei (2014)^20^	• Diagnosed patients, outpatients (n=63), 48 people under treatment • No control	PSQI	• There was no relationship between disease activity and sleep disorder (P=0.7).
Palagini (2014)^1^	• Diagnosed patients, outpatients (n=81), 71 people under treatment • Women with other chronic diseases (n=53)	PSQI, Insomnia Severity Index (ISI)	• ISI was 6.2±7.7 in the case group and 5.4±6.6 in the control group (P=0.4). • PSQI was 7.2±3.6 in the case group and 5.4±6.7 in the control group (P=0.0004). • The frequency of poor sleep quality was 65.4% in the case group and 39.6% in the control group (P=0.003). • The poor sleep quality in patients with lupus (OR: 2.5) was not significant in the regression model after the exclusion of depression.
Mirbagher (2016)^8^	• Diagnosed patients, outpatients (77), 60 people under treatment • The control group is the general population	PSQI	• Global PSQI was 7.06±0.46 in the case group and 5.68±3.43 in the control (P=0.001), which was significant in most of the PSQI subunits • SLEDAIs of the patients with good sleep and poor sleep were 2.5±3.1 and 3.6±4.0, respectively (P=0.2) • Patient activity (SLEDAI) was one of the predictors of poor sleep (beta=1.10, P=0.05)
Present study	• Patients newly diagnosed with lupus, outpatients (n=14) • Healthy volunteers (n=14)	Polysomnogram	• The frequency of sleep disorder in the case and control groups were 64.3% (n=9) and 50% (n=7), respectively (P=0.4) • SLEDAI was not associated with sleep-related items (r=0.459, P=0.01)

PSQI: Pittsburgh Sleep Quality Index; SLEDAI: systemic lupus erythematosus disease activity index; SLE: systemic lupus erythematosus; PML: progressive multifocal leukoencephalopathy.

Lack of any difference between the lupus patients and control group in terms of sleep disorders in the present study, can be explained as the result of the fact that our patients were newly diagnosed lupus cases, and had not received any medical treatment. It seems that the chronic consumption of corticosteroids can induce these disorders in this group of patients. Another reason might be related to the high frequency of sleep disorders in the general population of Iran, because the incidence of sleep disorders is highly dependent on geographical location, country under study, the age group.^15,16^

Iranian studies have presented that sleep disorders prevalence in Iran is in the range of 40–50%,^17,18^ which is similar to the frequency of sleep disorders obtained for healthy subjects in this research. In addition, it should be noted that sleep disorders are potentially higher in women than in men and even in the general population.^19^ Although nearly two-thirds of patients with lupus in this study were suffering from sleep disorders, there was no significant difference in the prevalence of these disorders among the general population.

In the current study, the mean serum melatonin level was not significantly different between the case and control groups. In the patients with lupus, only sleep latency to N1 and SAI had a significant positive correlation with the serum melatonin level. Possibly, the inappropriate quality of the two above-mentioned items in lupus patients caused a compensatory increase in melatonin. The results presented no significant correlation between the levels of melatonin and any of the sleep items in the control group.

To the best of our knowledge, only one study has investigated melatonin in patients with lupus. This carried out by Robeva et al. on 111 women with lupus and 46 healthy women, the daily melatonin levels in the lupus patients were significantly lower than those of the normal population. However, no such difference was observed in the present study. In the current research, we measured the nocturnal melatonin level, which is the right time to evaluate this hormone level.

Robeva et al. reported an indirect relationship between melatonin level and SLEDAI.^5^ However, such a relationship was not observed in the present study. Sleep disorders in the patients with lupus often present an increase in sleep latency, sleep fragmentation, arousal number, and sleep deprivation (associated with an increase in the stage one of the sleep), and a decrease in deep sleep in stages 3–4.^7^ Our results showed a positive relationship between melatonin levels and sleep latency to N1 in patients, but the role of melatonin in the pathogenesis of sleep disorder in patients with lupus should be more investigated.

The results of the present research and other studies regarding sleep disorders and prolactin level in lupus disease have been summarised in **Table 5** and **Table 6** respectively. Based on the results, in the case group, prolactin level was higher compare to the normal subjects. Nonetheless, this difference was not statistically significant. The results also revealed no significant correlation between the serum prolactin levels and any of the sleep factors.

**Table 6.** Comparison of the results of the present research with those of other studies regarding the role of prolactin in systemic lupus erythematosus.

**Author name**	**Research units (n)**	Results (prolactin^*^)
Rezaieyazdi and Hesamifard (2006)^21^	Patients diagnosed with lupus (n=30)	• Increased level of prolactin was observed in 33.3% of patients (10 of 30 patients). • Significant positive correlation between serum prolactin levels and disease activity (r=0.675, P<0.001).
Zakeri (2010)^22^	• Female patients (n=40) with active (n=12) and inactive (n=28) disease • Control group: healthy women (n=40)	• Hyper-prolactinaemia was observed in two cases in normal subjects and seven cases in the patients (N/S) • The hyper-prolactinaemia was observed active in six cases with active and six cases with inactive disease (P=0.001).
Paraiba (2010)^23^	• Patients diagnosed with lupus (n=30) • Healthy people (n=10)	• The mean prolactin levels were 6.4, 7.65, and 10.85 in healthy subjects, inactive lupus patients, and active lupus cases, respectively (P=0.01).
IQBAL (2011)^24^	• Patients with lupus (n=35) • Healthy people (n=35)	• The mean prolactin level was 9.92±0.70 in the healthy subjects and 65.34±24.22 in the lupus patients (P=0.001)
Ugarte-Gil (2014)^25^	• Patients diagnosed (n=160)	• The frequency of hyper-prolactinaemia was 29.4 % (n=47) in the patients. • There was a significant relationship between prolactin levels and severity of lupus damage (r=0.29, P<0.001)
Legorreta-Haquet (2016)^26^	• Female patients (n=26) with active (n=13) and inactive (n=13) disease • Control group: healthy women (n=17)	• Lymphocyte prolactin receptor levels were 34.44, 1931.50, and 3202.00 in the healthy subjects, inactive lupus cases, and active lupus patients, respectively.
Yang (2016)^27^	• Patients with lupus (n=30) • Healthy people (n=25)	• The mean prolactin level was mIU/L 580.9±419.9 mlU/l in the patient group and mIU/L 346.0±227.6 mlU/l in the healthy subjects (P=0.05). • Active disease was mIU/L 706.1±485.5, and inactive disease was mIU/L 393.2±191.3 (P=0.05). • There was a significant association between anti-dsDNA and prolactin level (r=0.397, P<0.05)
Elera-Fitzcarrald (2016)^28^	• Patients with lupus (n=185)	• The prolactin level of patients was 18.9. • The frequency of hyperprolactinemia in patients was 20%.
Song and Lee (2017)^6^*	• Patients with lupus (n=1,056) • Healthy people (n=426)	*Prolactin level was significantly higher in the SLE group than in the control group. *The SLE group in the Asian, Latin American, and mixed populations had significantly elevated prolactin level. *There was a significant positive correlation between circulating prolactin level and SLE activity.
Present study	•Patients with newly diagnosed lupus (n=14) • Healthy volunteers (n=14)	• The serum prolactin level was 18.02±10.70 in the patients and 14.57±8.40 in the control (P=0.3). • The serum prolactin level and SLEDAI had no relationship (P=0.4). • There was no correlation between the serum prolactin level and anti-dsDNA ratio (P=0.6), C3 ratio (P=0.1), and C4 ratio (P=0.6).

SLEDAI: Systemic lupus erythematosus disease activity index; SLE: systemic lupus erythematosus.

* Prolactin unit in all cases is ng/ml except the Yang(2016).

** Meta-analysis; #(>25 ng/ml in females and >16 ng/ml in males).

The strength of this study included the selection of newly diagnosed patients at the onset of the disease, the application of polysomnography to test the sleep disorders (as a tool with high accuracy), and simultaneous monitoring of the serum levels of hormones by performing the sleep test. Moreover, the control group was selected carefully with the same socioeconomic status. Exclusion of participants with other important emotional confounders was another important strength point of our study. One limitation of this study was the adoption of a relatively small sample size due to the problems with polysomnography.

## CONCLUSION

Regarding the presented results, more than half of the newly diagnosed lupus patients were suffering from sleep disorders, especially sleep apnoea. However, there was no significant difference between the lupus patients and healthy controls in this regard. Meanwhile, other studies have reported a higher incidence of sleep disorders in patients with lupus than in the healthy subjects. This study showed that the difference was not significant at least at the onset of the disease. Therefore, it can be concluded that the lupus disease has no significant effect on the pathogenesis of sleep disorders in the early phases of the disease. Our results are inconsistent with previous studies, probably due to the difference in disease duration. It seems that the chronicity and complications of the disease, as well as the adoption of glucocorticoid therapy for the chronic disease affect sleep quality in SLE patients more than disease duration.
